# Common Dermatoses in Children Referred to a Specialized Pediatric Dermatology Service in Mexico: A Comparative Study between Two Decades

**DOI:** 10.5402/2012/351603

**Published:** 2012-10-14

**Authors:** Blanca Rosa Del Pozzo-Magaña, Alejandro Lazo-Langner, Pedro Gutiérrez-Castrellón, Ramón Ruiz-Maldonado

**Affiliations:** ^1^Department of Pediatric Dermatology, Instituto Nacional de Pediatría, 04530 Mexico City, DF, Mexico; ^2^Division of Children's Health and Therapeutics, Children's Health Research Institute, London, ON, Canada N6C 2V5; ^3^Department of Medicine, University of Western Ontario, London, ON, Canada N6A 3K7; ^4^Department of Epidemiology and Biostatistics, University of Western Ontario, London, ON, Canada N6A 3K7; ^5^Clinical Research Department, Instituto Nacional de Pediatría, 04530 Mexico City, DF, Mexico

## Abstract

*Background*. Skin diseases among pediatric patients differ from those in adults. Epidemiological studies are scarce, and those performed in Mexican population date back thirty years. It is likely that these diseases might have changed their frequency. *Material and Methods*. Retrospective study in first-time patients referred to a pediatric dermatology service between January 1994 and December 2003. Demographics and diagnosis were recorded and compared with the results of a previous study performed in the same institution. *Results*. We included 5250 patients (52.55% female, 47.47% male) with 6029 diagnoses. The most frequent dermatoses found were atopic dermatitis (14.59%), viral warts (6.62%), acne (5.53%), pityriasis alba (3.98%), melanocytic nevi (3.85%), xerosis (3.57%), keratosis pilaris (3.19%), seborrheic dermatitis (2.37%), hemangioma (2.26%), and papular urticaria (2.24%). Most dermatoses increased their frequency when compared to the previous study. *Conclusion*. The frequency of pediatric dermatoses in our institution has changed in the last two decades. Environmental and sociocultural factors and institutional policies might account for these results.

## 1. Introduction

Skin diseases represent between 6% and 24% of pediatric consultation [1–3]. Skin diseases epidemiology in children and adolescents differ from that in adult patients because several diseases are exclusive to childhood. In addition, their manifestations, diagnosis, and treatment sometimes are also different. There are few studies on the epidemiology of skin diseases in children, and even fewer have explored timely changes in the patterns of such diseases [3–10]. In spite of the fact that such studies have been conducted in countries with different ethnic, environmental, and socioeconomic conditions, some dermatoses are frequent in all populations, such as infectious (warts and impetigo) [4, 10], atopic dermatitis [5, 11], genetically determined (keratosis pilaris) [6, 7], parasitic (papular urticaria, scabies, and pediculosis) [9], environment related (pityriasis alba) [9], and acne [9]. 

The frequency of skin diseases in a referral Mexican pediatric hospital population was first reported by our group in 1977 [12] in 10,000 patient referrals for skin conditions. In this study, the most common dermatoses in pediatric patients of 0–17 years old were papular urticaria (16.3%), atopic dermatitis (12.9%), viral warts (8.4%), impetigo (6.8%), pityriasis alba (6.6%), vitiligo (2.6%), acne (2.4%), diaper rash (2.0%), hemangiomas (1.8%), and melanocytic nevi (1.8%). It is worth noting that many diseases have been described only recently, and some others have decreased in frequency or have disappeared due to recent advances in prevention and treatment. We therefore sought to determine the current prevalence of skin diseases in children and adolescents attending the Dermatology Service of the Instituto Nacional de Pediatría in Mexico City, which is a large tertiary care referral center serving a population catchment area of approximately eighteen million. The secondary objective was to compare the current prevalence with the one previously reported in our institution [12] to evaluate changes in the patterns of skin diseases.

## 2. Methods

We conducted a retrospective analysis of all consecutive patients between 0 and 18 years of age that were referred for the first time to the Dermatology Service of the Instituto Nacional de Pediatría in Mexico City between January 1994 and December 2003. Information on age, gender, and diagnosis was collected from the files and consultation registry of our service. Patients with incomplete information were excluded from the study. Information was introduced in a computerized database for posterior analysis. Patients were grouped according to gender and age group divided as follows: newborns (<1 month), infants (1–12 months), toddlers (13–24 months), prescholars (25 months–6 years), scholars (>6–12 years), and adolescents (>12–17 years).

### 2.1. Statistical Analysis

The frequencies of specific diagnoses were grouped by age and sex and compared to the frequency previously reported in similar age groups [12], using 2-by-2 contingency tables. The proportions were compared using a chi-squared test with Yates' correction. A *P* value <0.05 was considered statistically significant.

## 3. Results

Between January 1994 and December 2003, there were 9,322 patients referred for the first time to the Dermatology Service of the Instituto Nacional de Pediatría, which represented 5.9% of the 157,304 first-time referrals to specialty services in our institution. We included in the study 5,250 patients of which 2491 (47.44%) were male, and 2759 (52.55%) were females. Some patients had more than one skin disease, and the total number of diagnoses was 6029. The distribution according to age group was as follows: newborns 51 (0.97%), (29 males, 22 females), infants 379 (7.21%), (191 males, 188 females), toddlers 420 (8%), (205 males, 215 females), pre-scholars 1304 (24.8%), (675 males, 629 females), scholars 2570 (48.95%), (796 males, 887 females), and adolescents 2750 (26.91%), (595 males, 818 females). In this study, 4072 patients were not included because they did not have complete information or diagnosis was not reported.

The more common diagnoses in the whole group and distributed by age and gender are shown in Tables 1, 2, and 3 together with the comparison with the frequency reported in the previous study [12]. In the whole population, the most frequent dermatosis was atopic dermatitis (14.6%) followed by viral warts (6.6%), acne (5.5%), pityriasis alba (4.0%), and melanocytic nevi (3.8%). All these conditions had a statistically significant increase in their frequency compared to the previous study (*P* < 0.01); on the contrary, there was a significant decrease in the frequency of papular urticaria (16.3% versus 2.24%; *P* < 0.01) (Table 1).

No significant changes were observed in the frequency of dermatoses in newborns. In infants, toddlers, prescholars, and scholars, there was a change in the most frequent dermatoses; in the previous study, the most frequent diagnoses were infectious or parasitic, whereas in the present study, atopic dermatitis was the most frequent disease in these age groups. Acne continued to be the most frequent cause of consultation in adolescents (Table 2). In addition, some differences were observed between genders. Seborrheic dermatitis, papular urticaria, and miliaria were more frequent among males, whereas hemangiomas and scars were more frequent among females (Table 3).

## 4. Discussion

The data in the present work is representative of the Mexican pediatric population referred to a service specialized in pediatric dermatology; however, it is necessary to consider that many common skin diseases, such as diaper rash and chickenpox, are frequently diagnosed and treated in the primary care setting or in other specialized services, which might result in a lower observed frequency within a specialized dermatology service when compared to open population.

Atopic dermatitis was the most frequent dermatosis among all age groups, and compared to the previous study, we observed a significant increase in the prevalence of atopic dermatitis in infants, toddlers, prescholars, and scholars. We believe that this is due to the fact that atopic dermatitis is observed and/or diagnosed early. It is also worth noting that the most frequent dermatoses in these age groups in the previous study were parasitic; therefore, it is possible that improvement in sanitation, housing, and socioeconomic status might have contributed to these changes. Such changes are most likely a reflection in the national gross domestic product per capita which increased more than four times between 1975 and 2003. Warts are a common dermatosis around the world. The frequency of warts in our study was similar to that in other studies [4] conducted in otherwise healthy population. This is interesting because since our hospital is a national referral center, a substantial proportion of our patients have associated diseases which can affect immunity. The lack of difference between our study and other previously published works might suggest that in our patients such diseases were not a predisposing factor for warts; however, because information about other diseases in our children was unavailable, this is still speculative. It is important to mention that awareness about dermatologic problems has increased in many services in this hospital resulting in more frequent referrals. 

Pityriasis alba was the fourth most frequent dermatosis observed in our study although its prevalence in open population might be higher, and it might be underrepresented in our patients. However, a recent study conducted in a general practice setting in The Netherlands showed that in their population pityriasis alba was not a frequent diagnosis, and although the characteristics of their population might explain this difference, it is noteworthy that the study included a substantial proportion of nonwestern immigrants [10]. Hemangiomas are the most frequent benign tumors in children with an informed frequency of 2.5% in newborns and a male-to-female ratio of 3 to 1 [13]. In the present study, that hemangiomas represented 2.25% of dermatosis in the whole population and 12.96% in newborns with a male-to-female ratio of 2.88 to 1, similar to that previously described by other authors [13].

Not surprisingly, acne was the most frequent dermatosis among adolescents with no differences between genders or compared to the previous study. Contact dermatitis is more frequent in adult patients, since it is related to exposure to chemicals; notwithstanding this, its frequency in our population was 1.48% with the adolescents and scholars being the most affected groups. Neurofibromatosis presents in 1/(3,000–4,000) population [14]. In our work, we observed it in 1.39% which was higher than the previous study. Postinflammatory hyperpigmentation was among the most frequent conditions in our study. Even though this is a sequel to other dermatoses and it is usually difficult to establish the initial diagnosis, it indicates that the skin of our patients which is usually dark has a higher predisposition to developing this change after inflammatory processes. Scars are also secondary processes, and they were the fourteenth place (1.64%). This group included patients with hypertrophic and keloid scars, secondary to surgery, burns, acne, and other conditions. The higher frequency that was observed compared to the previous study might be explained because in recent years our hospital conducted studies for scar treatment. Dermatophytoses were the eighteenth most frequent dermatosis in our patients (1.39%). Even though there was a significant decrease in dermatophytoses as a group compared to the previous study, this was due to a decrease in *tinea capitis *(16% in our study), whereas the cases of *tinea pedis* (58.6% in our study) actually increased in adolescents and scholars, probably due to changes in footwear. *Tinea corporis*-*tinea cruris* comprised 25.3% of the total cases of dermatophytoses. Vascular malformations comprise a vast group of alterations characterized by the presence of aberrant vessels. In our study, these were the twentieth most frequent dermatosis. Its incidence, as well as that of hemangiomas, was higher than that reported by other groups probably because our institution is a referral center for these conditions.

Some potential limitations to our work should be noted. First, even if the number of patients is large, a substantial proportion of them had to be excluded because of incomplete information. Additionally, due to the retrospective nature of the study, it was not possible to analyze variables that could have been important such as comorbid conditions. Nevertheless, the information obtained is invaluable to realize that there have been important changes in the frequency of dermatoses in our population since the previous study. The epidemiology of skin diseases at the Instituto Nacional de Pediatría in Mexico City has modified in the last 30 years. Atopic dermatitis is currently the most frequent pathology, whereas the infectious and parasitic diseases have decreased significantly. These observations are consistent with similar studies in other countries [10]. Changes are probably related to improvement in sanitation and access to health services, and also environmental and weather variation might have played an important role. It is also possible that modifications in the institutional policies might be related to the findings. The observed changes suggest that it is necessary to modify the current strategies for education, prevention, and health care practice in order to provide patients with an optimal management.

## Figures and Tables

**Table 1:** Most frequent dermatoses in children of 0–18 years.

Diagnosis	1971–1975 *N* (%)	1994–2003 *N* (%)	*P *
Atopic dermatitis	1391 (12.9)	879 (14.6)	0.01
Warts	916 (8.4)	399 (6.6)	<0.01
Acne	268 (2.5)	333 (5.5)	<0.01
Pityriasis alba	718 (6.6)	240 (4.0)	<0.01
Melanocytic nevi	200 (1.8)	232 (3.8)	<0.01
Xerosis	101 (0.9)	215 (3.6)	<0.01
Keratosis pilaris	109 (1.0)	192 (3.2)	<0.01
Seborrheic dermatitis	109 (1.0)	143 (2.4)	<0.01
Hemangioma	203 (1.8)	136 (2.3)	0.10
Papular urticaria	1766 (16.3)	135 (2.2)	<0.01
Miliaria	182 (1.7)	118 (1.9)	0.20
Postinflammatory hyperpigmentation	NR	105 (1.7)	—
Alopecia areata	94 (0.9)	100 (1.7)	<0.01
Scars	37 (0.3)	99 (1.6)	<0.01
Vitiligo	281 (2.6)	90 (1.5)	<0.01
Contact dermatitis	165 (1.5)	89 (1.5)	0.36
Neurofibromatosis	86 (0.8)	84 (1.4)	<0.01
Dermatophytosis	261 (2.4)	84 (1.4)	<0.01
Vascular malformation	NR	76 (1.3)	—
Insect bite	NR	74 (1.2)	—

*N*: number of patient referrals; NR: not reported; —: not estimable.

**Table 2:** Most frequent dermatoses by age group.

Diagnosis	1971–1975 *N* (%)	1994–2003 *N* (%)	*P *
Newborns (<1 month)			
Miliaria	13 (22.4)	10 (18.5)	0.10
Hemangioma	4 (6.9)	7 (12.9)	0.38
Lymphatic malformations	NR	6 (11.1)	—
Melanocytic nevi	2 (3.4)	3 (5.6)	0.58
Impetigo	NR	2 (3.7)	—
Seborrheic dermatitis	7 (12.0)	2 (3.7)	0.23
Epidermal cyst	NR	2 (3.7)	—
Postinflammatory hyperpigmentation	NR	2 (3.7)	—
Vascular malformations	NR	2 (3.7)	—
Mongolian spot	NR	2 (3.7)	—
Infants (1–12 months)			
Atopic dermatitis	76 (13.2)	89 (20.5)	<0.01
Hemangioma	121 (9.0)	57 (13.2)	0.01
Miliaria	120 (9.0)	42 (9.7)	0.16
Diaper rash	133 (10.0)	23 (5.3)	<0.01
Seborrheic dermatitis	56 (4.2)	20 (4.6)	0.71
Vascular malformations	NR	16 (3.7)	—
Scabies	215 (16.1)	10 (2.3)	<0.01
Neurofibromatosis	NR	9 (2.1)	—
Candidiasis	24 (1.8)	9 (2.1)	0.71
Nevus anemicus	NR	8 (1.8)	—
Toddlers (12–24 months)			
Atopic dermatitis	160 (12.0)	114 (22.4)	<0.01
Seborrheic dermatitis	NR	48 (9.4)	—
Miliaria	35 (2.6)	33 (6.5)	<0.01
Diaper rash	51 (3.8)	29 (6.9)	0.01
Papular urticaria	457 (34.2)	22 (5.2)	<0.01
Hemangioma	29 (2.1)	22 (5.2)	<0.01
Xerosis	NR	13 (2.6)	—
Urticaria	NR	11 (2.2)	—
Insect bites	NR	9 (1.8)	—
Vascular malformations	NR	9 (1.8)	—
Prescholars (24 months–6 years)			
Atopic dermatitis	448 (15.4)	287 (19.9)	<0.01
Xerosis	NR	80 (5.6)	—
Warts	139 (4.8)	67 (4.7)	0.82
Papular urticaria	735 (25.3)	65 (4.5)	<0.01
Melanocytic nevi	NR	49 (3.4)	—
Pityriasis alba	182 (6.2)	46 (3.2)	0.05
Postinflammatory hyperpigmentation	NR	32 (2.2)	—
Alopecia areata	NR	30 (2.1)	—
Miliaria	NR	27 (1.9)	—
Vitiligo	52 (1.8)	36 (1.8)	0.99
Scholars (>6–12 years)			
Atopic dermatitis	432 (11.7)	262 (13.7)	0.37
Warts	480 (13.0)	199 (7.7)	<0.01
Pityriasis alba	381 (10.3)	112 (5.9)	<0.01
Melanocytic nevi	NR	94 (4.9)	—
Xerosis	NR	84 (4.4)	—
Keratosis pilaris	NR	84 (4.4)	—
Scars	NR	42 (2.2)	—
Alopecia areata	NR	41 (2.1)	—
Postinflammatory hyperpigmentation	NR	38 (2.0)	—
Papular urticaria	358 (9.7)	35 (1.8)	<0.01
Adolescents (>12–18 years)			
Acne	244 (15.9)	298 (17.8)	0.15
Warts	190 (12.4)	129 (7.7)	<0.01
Atopic dermatitis	174 (11.5)	127 (7.6)	<0.01
Keratosis pilaris	34 (2.2)	77 (4.6)	<0.01
Pityriasis alba	127 (8.3)	76 (4.5)	<0.01
Melanocytic nevi	NR	75 (4.5)	—
Scars	NR	38 (2.3)	—
Dermatophytosis	29 (1.9)	36 (2.2)	0.59
Xerosis	NR	33 (2.0)	—
Onychomycosis	NR	33 (2.0)	—

*N*: number of patient referrals; NR: not reported; —: not estimable.

**Table 3:** Most frequent dermatoses by gender 1994–2003.

Diagnosis	Male *N* (%)	Female *N* (%)	*P *
Atopic dermatitis	415 (14.5)	464 (14.7)	0.88
Warts	186 (6.5)	213 (6.7)	0.75
Acne	165 (5.8)	168 (5.3)	0.47
Pityriasis alba	105 (3.7)	135 (4.3)	0.26
Melanocytic nevi	103 (3.6)	129 (4.1)	0.36
Xerosis	99 (3.5)	116 (3.7)	0.71
Keratosis pilar	84 (2.9)	108 (3.4)	0.32
Seborrheic dermatitis	86 (3.0)	57 (1.8)	<0.01
Hemangioma	35 (1.2)	101 (3.2)	<0.01
Papular urticaria	78 (2.7)	57 (1.8)	0.02
Miliaria	74 (2.6)	44 (1.4)	<0.01
Postinflammatory hyperpigmentation	57 (2.0)	48 (1.5)	0.19
Alopecia areata	51 (1.8)	49 (1.5)	0.54
Scars	32 (1.1)	67 (2.1)	<0.01
Vitiligo	46 (1.6)	44 (1.4)	0.55
Contact dermatitis	38 (1.3)	51 (1.6)	0.42
Neurofibromatosis	52 (1.8)	32 (1.0)	0.10
Dermatophytosis	44 (1.5)	40 (1.3)	0.43
Vascular malformation	40 (1.4)	36 (1.1)	0.43
Insect bite	41 (1.4)	33 (1.0)	0.21

*N*: number of patient referrals.
